# Synthesis of anthradithiophene containing conjugated polymers *via* a cross-coupling strategy†

**DOI:** 10.1039/d0ra09195b

**Published:** 2021-01-04

**Authors:** Waseem A. Hussain, Kyle N. Plunkett

**Affiliations:** Department of Chemistry and Biochemistry, Southern Illinois University Carbondale Illinois 62901 USA kplunkett@chem.siu.edu

## Abstract

New conjugated polymers that incorporate dihexylanthradithiophene (DHADT) in the main chain were prepared by Stille, Sonogashira, and Yamamoto cross-coupling polymerization reactions. The polymerization chemistry is enabled by a soluble 5,11-dibromodihexylanthradithiophene monomer that is capable of cross-coupling reactions. Five readily soluble DHADT containing polymers were prepared and characterized experimentally and computationally. These polymers possess HOMO energies of −5.18 eV to −5.43 eV and LUMO energies of −3.0 eV to −2.82 eV. The notable optical features include broad absorption and band gaps ranging from 1.62 eV to 2.15 eV. Polymers were tested in organic field effect transistors and were found to operate in the p-type regime.

## Abstract 2

Incorporation of anthradithiophene (ADT) into conjugated polymers *via* cross-coupling reactions is enabled by a soluble halogenated ADT precursor.

## Graphical abstract

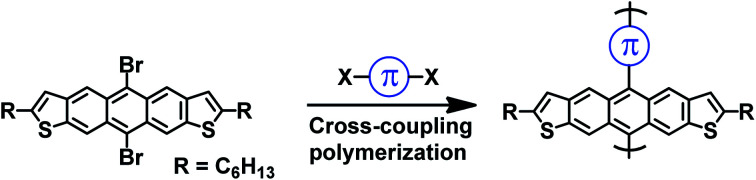

## Introduction

Pentacenes and their isoelectronic heteroacene analogues have long been investigated for their applications as optoelectronic materials in organic field effect transistors, organic photovoltaics and organic light emitting diodes.^1–5^ Despite pentacene-based materials having high hole carrier mobilities, their poor photostability and solution processability have limited their applications in conjugated polymer materials and have led to the investigation of alternative thiophene-fused polycyclic aromatic hydrocarbons. Anthradithiophenes (ADTs) in particular have been developed for their isoelectronic character with pentacenes.^6–11^ ADTs have shown promise owing to their higher stability and solubility compared to pentacenes.^12^ The lowered HOMO levels are owed to the peripherally fused thiophene rings that impart greater stability in ADT systems by inducing loss of aromaticity that hinders endo-peroxide formation at the central 5,11 positions of ADT.^6,13^ ADT derivatives with hydrocarbon side chains^6,14^ have imparted even more solubility and provide a potential building block for conjugated polymers. However, in relation to pentacene-based polymers,^15–17^ even fewer conjugated polymers containing the more stable ADT derivatives have been prepared.^18–22^

**Fig. 1:**
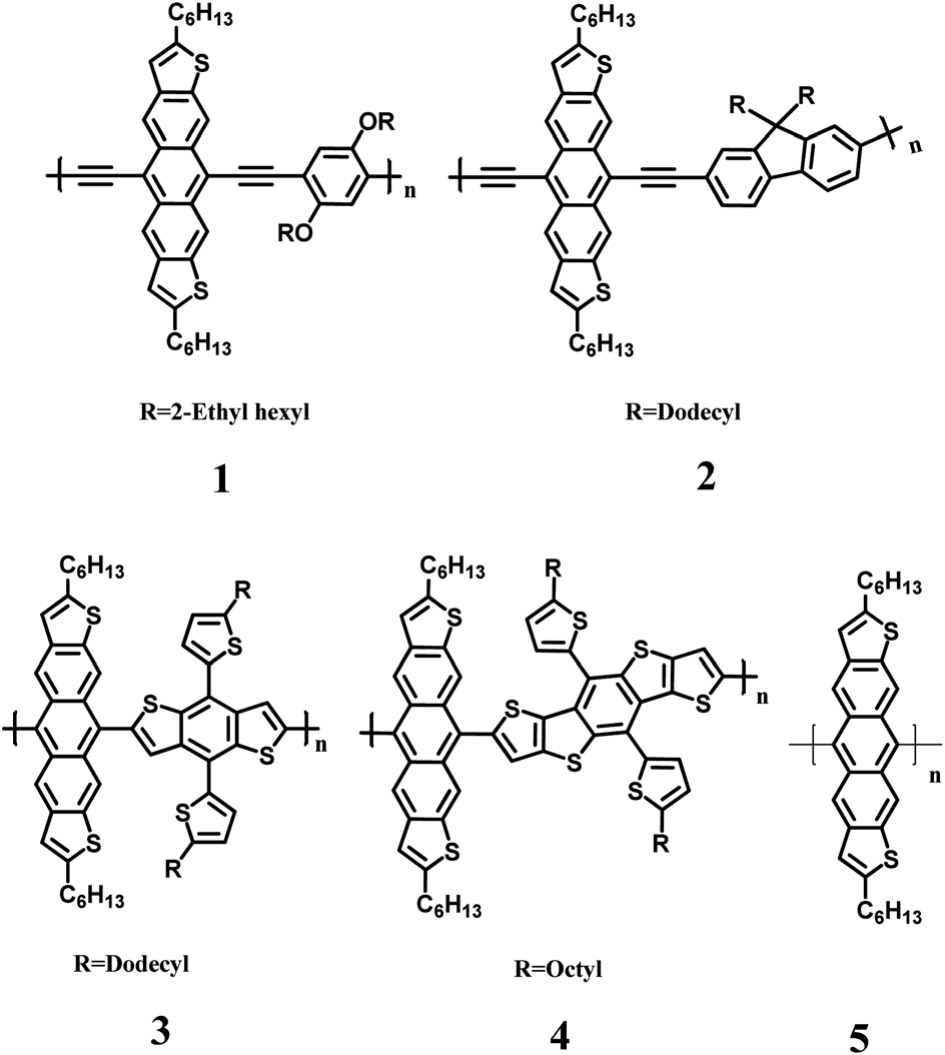
DHADT containing copolymers 1–5.

**Scheme 1:**
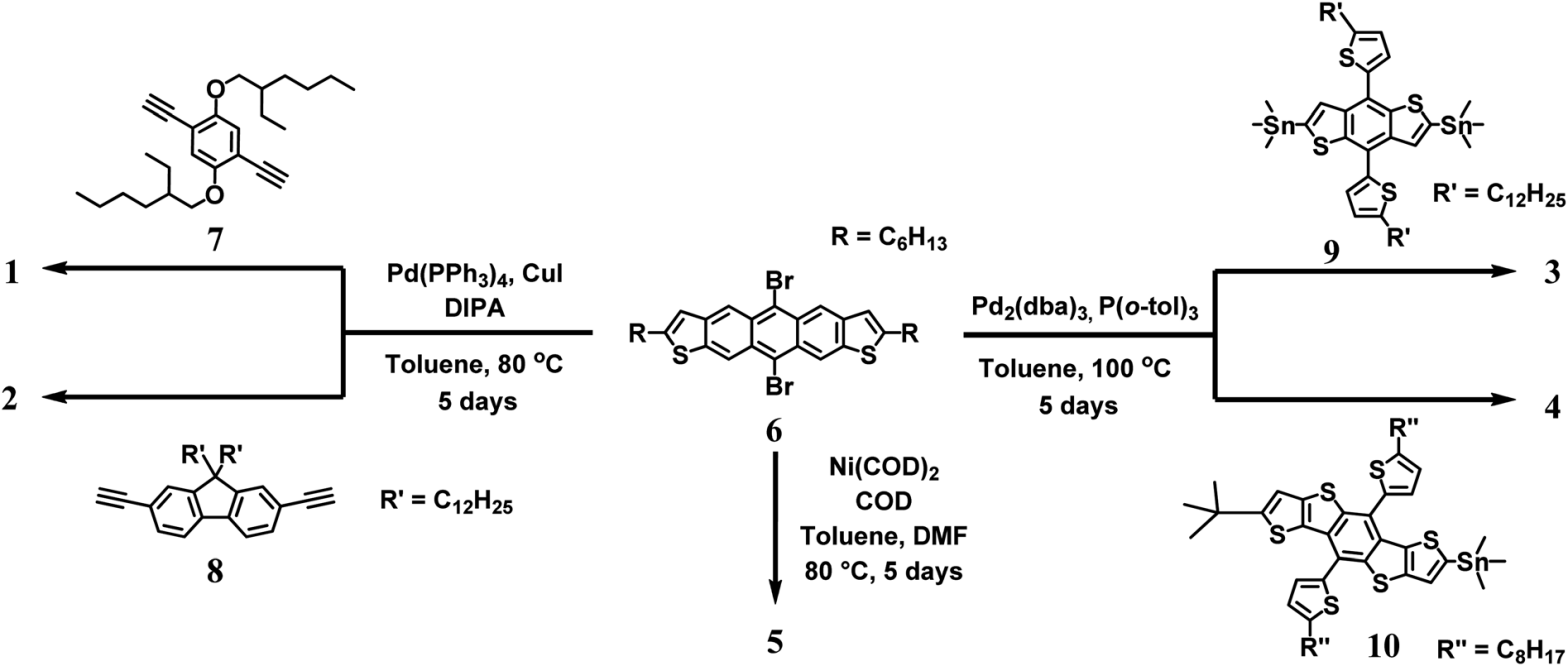
Synthetic route to DHADT fused conjugated co-polymers 1–5.

Here we report the synthesis of a new series of conjugated copolymers (1–5, Fig. 1) that are accessible through a metal catalyzed cross-coupling reaction at the 5 and 11 positions of dihexylanthradithiophene (DHADT). This strategy is different than the traditional method to create 5,11-functionalized ADT polymers, which rely on nucleophilic substitutions on anthradithiophene quinones.^23,24^ Furthermore, this linkage configuration allows for the incorporation of linear solubilizing side-chain that could help backbone packing and charge transport.^19^ Co-polymers 1 and 2 were prepared by a Sonogashira cross-coupling polymerization while copolymers 3 and 4 were prepared using Stille cross-coupling reactions of stannanes. A homopolymerization of DHADT was performed using Yamamoto cross-coupling conditions. These metal catalyzed transformations were possible owing to our recent report of a soluble and isomerically pure 5,11-dibromodihexylanthradithiophene 5 (Scheme 1).^25^ The halogenated ADT monomer provides alternative reaction pathways to enlarge the library of possible coupling partners and to expand the possible aryl substituents (*e.g.*, ester groups) that would not be compatible with the traditional nucleophilic substitutions pathway. An initial version of this work was deposited to ChemRxiv on July 23, 2020.^26^

## Results and discussions

Two new ethynylene containing polymers were prepared *via* a Sonogashira cross-coupling polymerization between 6 and two bis-ethynylene containing monomers, 1,4-bis((2-ethylhexyl)oxy)-2,5-diethynylbenzene 7 and 9,9-didodecyl-2,7-diethynyl-9*H*-fluorene 8 (Scheme 1). The polymerization employed the catalyst system of Pd(PPh_3_)_4_ and CuI in toluene and diisopropyl amine. Precipitates of sharp purple and dark maroon colored materials were obtained for polymers 1 and 2, respectively. We found that long reaction times of up to five days were required to access reasonable molecular weight polymers (*M*_n_ = 9–16 kDa, Table 1). Polymers 3 and 4 were synthesized with commercially available stannane monomers 9 and 10 by the employment of Stille coupling reaction conditions. The catalyst system employed Pd_2_(dba)_3_ with P(*o*-Tol)_3_ in toluene at 100 °C under argon for five days. The polymers were precipitated and filtered to afford 3 and 4 as brick red colored solids with molecular weights (*M*_n_ ∼ 4 kDa) that were less than the ethynylene containing derivatives. Lastly, the homopolymerization of 6 was carried out using Ni(COD)_2_ to afford low molecular weight oligomer/polymer 5. The lack of high molecular weight is most likely owing to the steric restrictions of the aryl–aryl coupling from the adjacent fused rings of ADT.

**Table 1:** Summary of molecular weights and optoelectronic properties of polymers 1–5^a^

	*M*_n_	*M*_w_	PDI	*E*_ox/onset_ (V)	*E*_red/onset_ (V)	HOMO (eV)	LUMO (eV)	E-chem (eV)	Optical gap (eV)
1	15 800	56 400	3.6	0.38	−1.80	−5.18	−3.00	2.18	1.62
2	8600	29 400	3.4	0.34	−1.80	−5.14	−3.00	2.14	1.84
3	4200	6900	1.7	0.65	−2.09	−5.45	−2.71	2.74	2.13
4	4000	7700	1.9	0.63	−1.98	−5.43	−2.82	2.61	2.15
5	2800	3900	1.3	0.46	−1.99	−5.26	−2.81	2.45	2.22

a Potentials are measured relative to a ferrocenium/ferrocene redox couple used as an internal standard (Fig. 3). *E*_ox/onset_ is the oxidation onset potential and *E*_red/onset_ is the reduction potential onset. The redox potential onsets were used to calculate HOMO and LUMO *via* ferrocene standard in vacuum (4.8 eV). The *M*_n_, *M*_w_ and polydispersity PDI values were measured with GPC with THF as eluent and polystyrene standard.

**Fig. 2:**
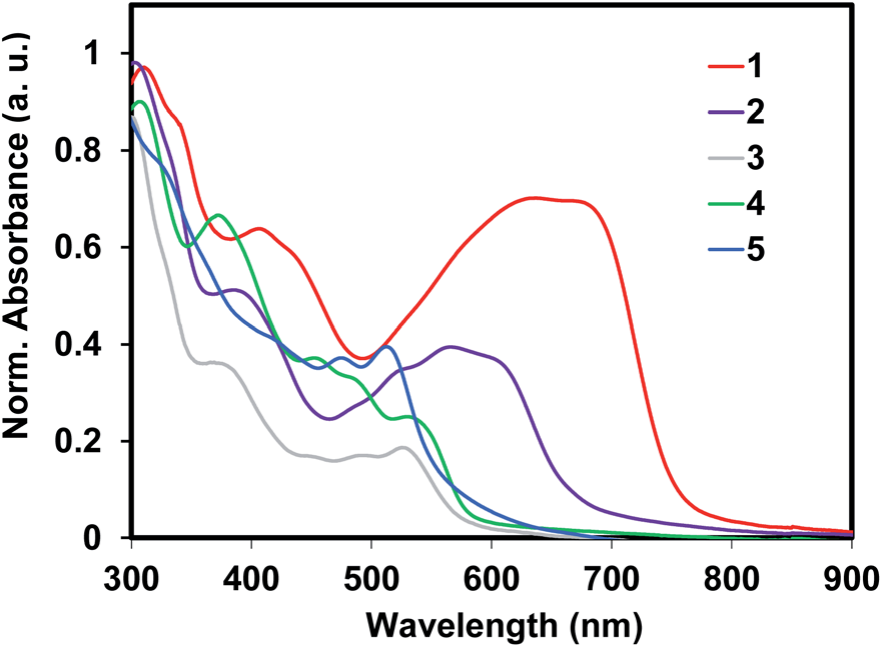
Diffused reflectance absorption spectra of polymers 1–5.

The diffused reflectance absorption spectra of polymers (1–5) are shown in Fig. 2. The optical gaps of the Sonogashira cross-coupled copolymers (1 and 2) were lower than the Stille cross-coupled copolymers (3 and 4) and to the homopolymer 5. While 1 and 2 had optical gaps of 1.62 eV and 1.84 eV; polymers 3, 4, and 5 possessed optical gaps of 2.13, 2.15, and 2.22 eV, respectively. This considerable difference suggests better interchain delocalization between monomers in 1 and 2. The steric encumbrance of the aryl–aryl linkages of 3–5 limits the coplanarity of the aromatic residues more so than the ethynylene containing polymers 1 and 2. Overall, these band gap values are smaller than the isoelectronic pentacene containing conjugated polymeric analogues^15^ and similar to other reported ADT containing polymers.^18,19^

**Fig. 3:**
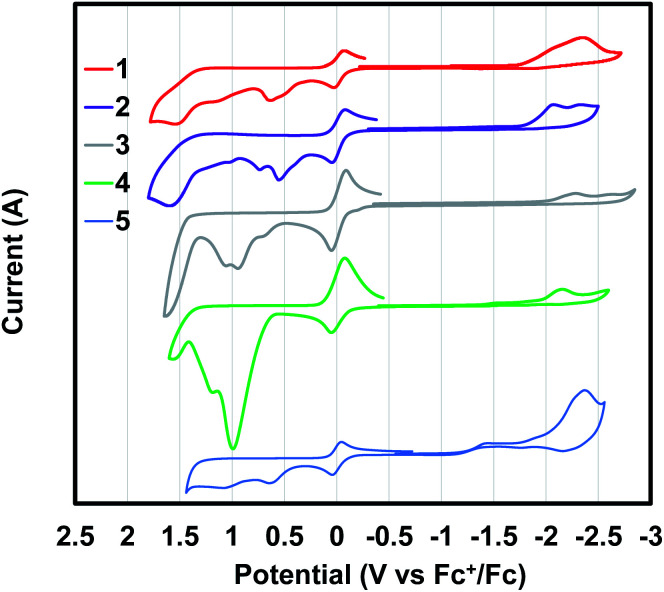
Cyclic voltammograms of thin films of polymer 1–5 in 0.1 M tetrabutylammonium hexaflurophosphate (TBAPF_6_) in acetonitrile with glassy carbon working electrode, platinum counter electrode and Ag/AgCl reference electrode. Scan rate of 100 mV s^−1^. Ferrocene was used as internal standard and referenced to 0 V.

Cyclic voltammetry (CV) of polymer films coated on a glassy carbon working electrode were measured in acetonitrile (Fig. 3). All oxidation and reduction transitions were irreversible for all polymers. Using the onset of oxidation and reduction in the CV, the highest occupied molecular orbitals (HOMO) and lowest unoccupied molecular (LUMO) orbitals were calculated using ferrocene as an internal standard (Table 1). The HOMO energies were found to vary between −5.14 eV and −5.45 eV. The LUMO energies were found to vary between −2.7 eV and −2.82 eV.

**Fig. 4:**
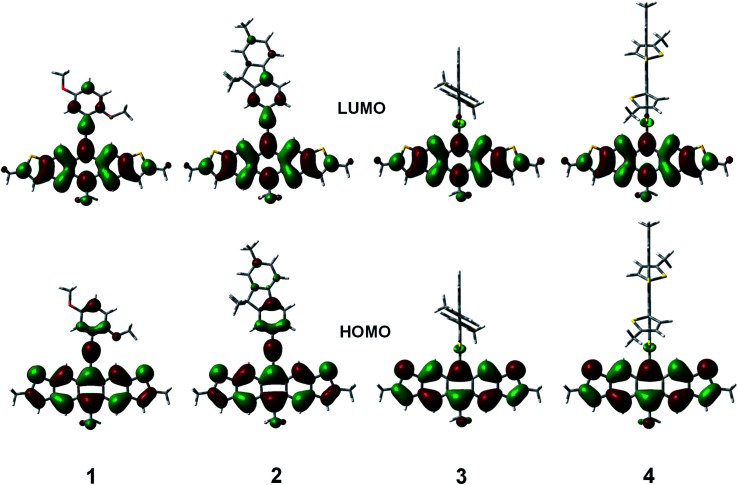
B3LYP/6-311g(d,p) calculated HOMO and LUMO contours polymers 1–4.

The geometries of the copolymer repeat units of 1–4 were optimized using density functional theory with the B3LYP functional and 6-311G(d,p) basis set (Fig. 4). Solvent free default convergence criteria were used to calculate the energy minimized structures. The optimized geometries suggest the HOMO and LUMO contours are largely concentrated on the DHADT unit with some delocalization at the adjoining monomers in the ethynylene containing polymers 1 and 2. Negligible probability for the HOMO or LUMO contours was found on the dithiophene monomers in copolymers 3 and 4. Furthermore, little frontier orbital overlap between adjacent ADT monomer units were found for the homopolymer 5 (ESI†). The minimized geometries confirm that the ethynylene containing polymers 1 and 2 can possess a more planarized structure and therefore enable significant overlapping of the frontier molecular orbitals (FMOs). In contrast, the monomers in the Stille copolymers 3 and 4 exhibit orthogonal geometry inhibiting the overlap of FMOs and lead to weak delocalization of the electronic cloud. These findings are substantiated by the diffused reflectance spectra (Fig. 2) of copolymer 1–4 showing low energy absorption onsets and broad band absorption for copolymer 1–2 and relatively higher energy onsets for copolymer 3 and 4. The structural optimization of copolymer units 1–4 was followed by the UV-vis spectra determination utilizing CAM-B3LYP/6-311G(d,p) (ESI†). The calculated *λ*_max_ values for the UV-vis spectra generally corroborated the experimental results. Copolymers 1 and 2 were found to be red-shifted in relation to copolymers 3 and 4.

Thin films of polymers 1–5 were prepared on octadecyltrichlorosilane coated silicon wafers (Si/SiO_2_) by spin coating from chloroform and were studied by tapping mode AFM (ESI†). The topological surfaces of all polymers show interconnected texture. Polymer 1 and 2 gave a more homogeneous structure with root mean square (RMS) surface roughness ranging from 0.333 nm to 0.402 nm, respectively. The RMS calculated based on variation of surface height suggests their topography to be nearly flat. The RMS roughness for polymer 3 and 4 was 6.15 nm and 2.14 nm, respectively, suggesting a more granular topography. The morphological characteristics of polymers seemed to have consistent association with their molecular weights. With the decreasing *M*_n_ values from polymer 1 to 4, the surface texture starts becoming granular from flat. Organic field effect transistors (OFETs) in a bottom gate, bottom contact configuration on silicon wafers were fabricated with all polymers. Polymer 1 as an active material gave the best performance with an average hole mobility of 2.2 × 10^−5^ cm^2^ V^−1^ s^−1^ (ESI†). Polymers 2 and 4 gave no OFET signal while polymer 3 gave a small signal with average hole mobility of 5.0 × 10^−7^ cm^2^ V^−1^ s^−1^. A limiting factor in these device performances may be attributed to the lower molecular weights of these derivatives.

In conclusion, we have synthesized two new classes of conjugated homopolymer and co-polymers incorporating DHADT. The dibrominated ADT monomer provides an alternative synthetic route to make soluble conjugated polymers. The higher molecular weight samples based on ethynylene chemistry gave better thin film properties including broader light absorption, more regular films, and better performing materials in OFETs.

## Conflicts of interest

There are no conflicts to declare.

## Supplementary Material

RA-011-D0RA09195B-s001
