# Supplementary material for: Synthesis of anthradithiophene containing conjugated polymers via a cross-coupling strategy
Source: RSC Adv. 2021 Jan 4;11(2):996–1000. doi: 10.1039/d0ra09195b (PMC8693243; doi:10.1039/d0ra09195b)
Supplement: RA-011-D0RA09195B-s001 [file RA-011-D0RA09195B-s001.pdf]

Electronic Supplementary Material (ESI) for RSC Advances.
This journal is © The Royal Society of Chemistry 2020

Synthesis of Anthradithiophene Containing Conjugated Polymers via a Cross-coupling Strategy

Waseem A. Hussain and Kyle N. Plunkett*

Department of Chemistry and Biochemistry, Southern Illinois University, Carbondale, Illinois 62901, United States

kplunkett@chem.siu.edu

CONTENTS: **PAGE**

1)	DFT calculated structures of **1-6**	SI2
2)	Solution UV-Vis and Calculated UV-Vis	SI3
3)	AFM	SI4
4)	Experimental Section	SI4-SI5
5)	NMR Spectra	SI6-SI9
6)	DFT Calculation Tables	SI10-SI20

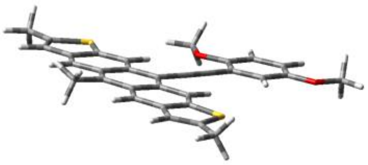

1

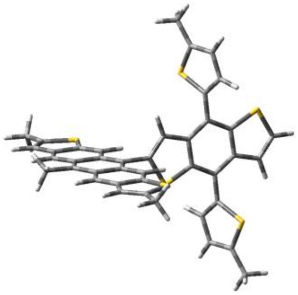

3

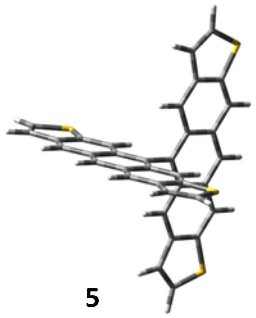

5

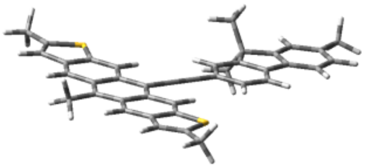

2

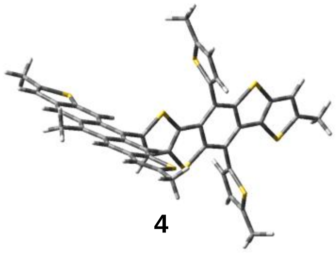

4

Figure S1. The B3LYP/6-311g(d,p) DFT minimized structures of polymers **1-5** (with methyl substituents replacing alkyl chains and polymeric connectivity in **1-4** and 'H' in **5**).

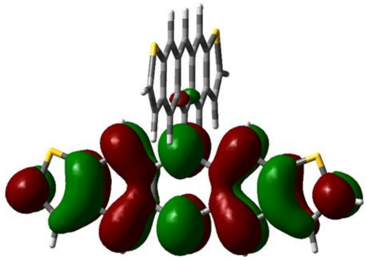

LUMO

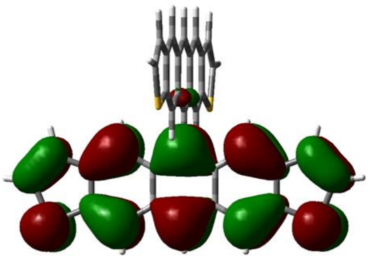

HOMO

Figure. S2 Homo and Lumo contours of Polymer **5** calculated using DFT B3LYP/6-311g(d,p).

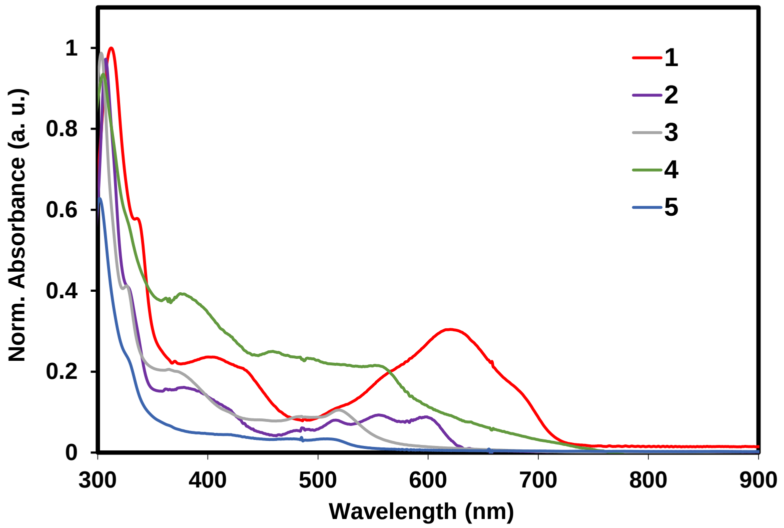

Figure S3. UV-Vis of polymers 1-5 in dichloromethane.

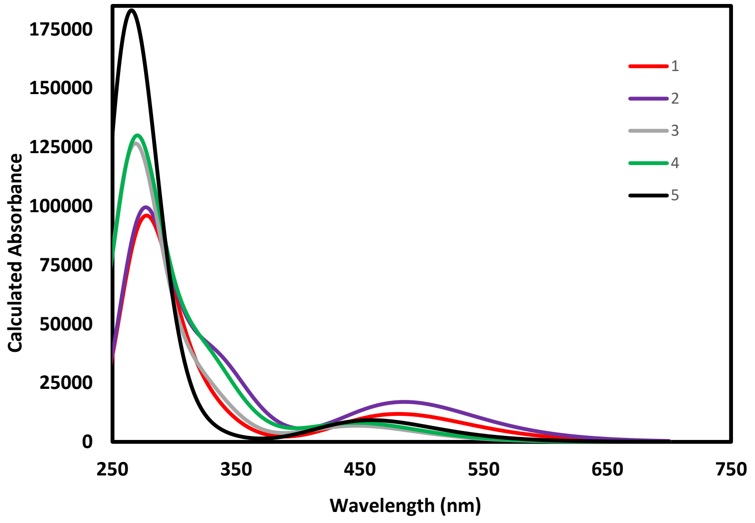

Figure S4. Calculated absorption spectra of polymers **1-5** using TD-DFT Cam-B3LYP/6-311G(d,p).

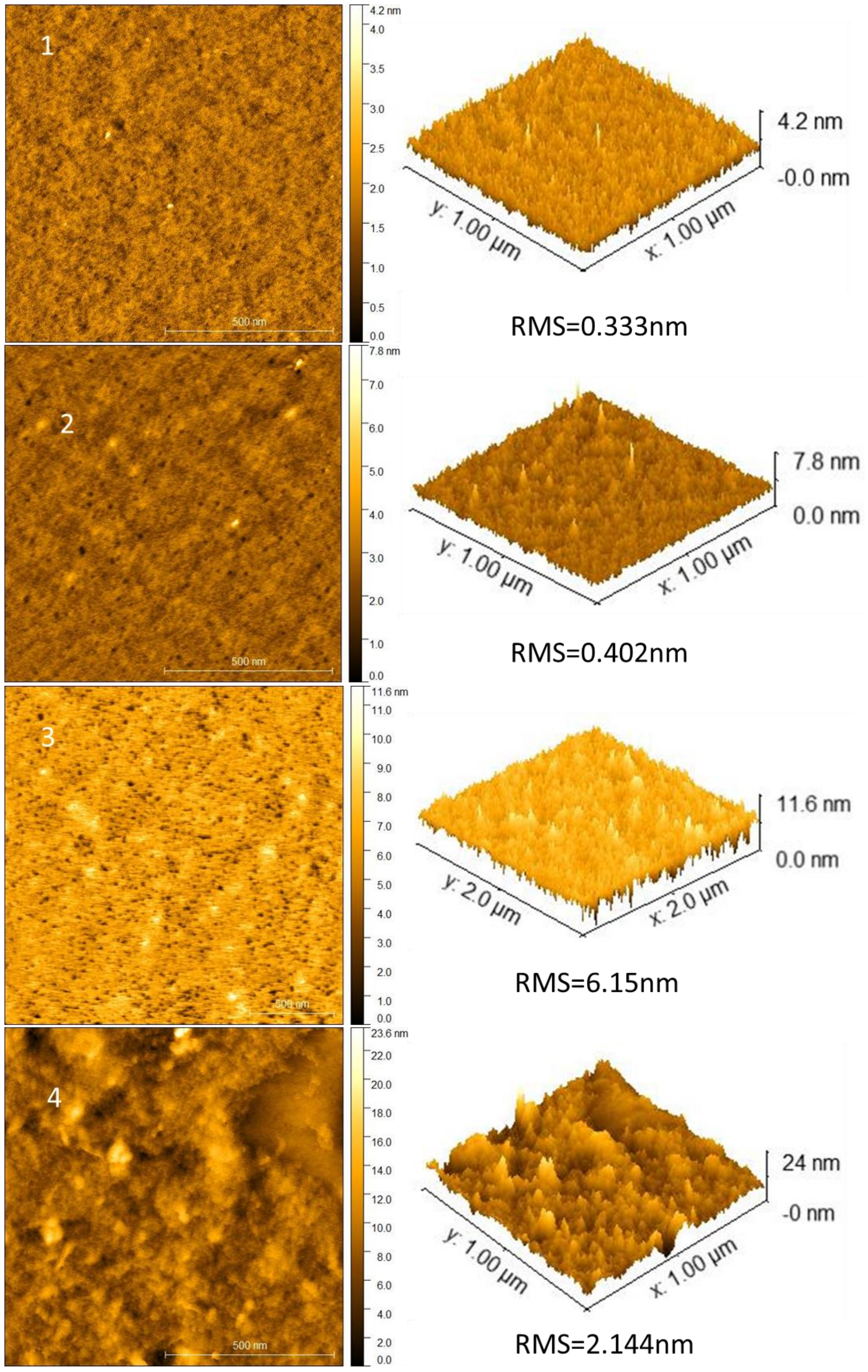

Figure S5. Tapping mode AFM of Polymer 1-4, with topography on the left and 3-d images on the right along with the calculated roughness (RMS) profiles.

OFET devices were prepared in a bottom contact, bottom gate configuration on octadecyltrichlorosilane (OTS) treated Si/SiO_2 wafer (Osilla). Active layers of **1-4** were prepared spin coating from a 1 mg/mL solution of polymer in chloroform. Channel widths were 30 μm and channel lengths were 1 mm. Current-voltage measurements were performed on a semiconductor characterization system in air.

Charge carrier mobility was extracted from the transfer characteristics in the saturation regime using:

$$\mu_{sat} = \frac{2L}{WC_i}\left(\frac{d\sqrt{I_D}}{dV_G}\right)^2$$

L = 1000 μm W = 30 μm C_i = 1.09 X10^{-8} F cm^{-2} $\frac{d\sqrt{I_D}}{dV_G}$ = slope of linear region

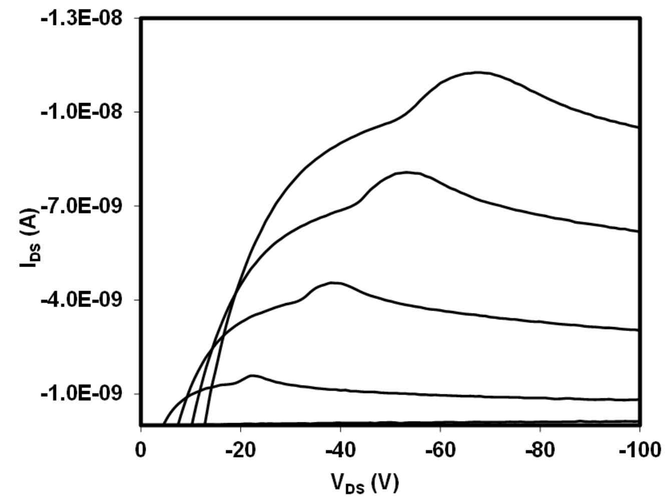

Figure S5=6. Typical output curves for polymer 1 exhibiting p-type semiconducting behavior.

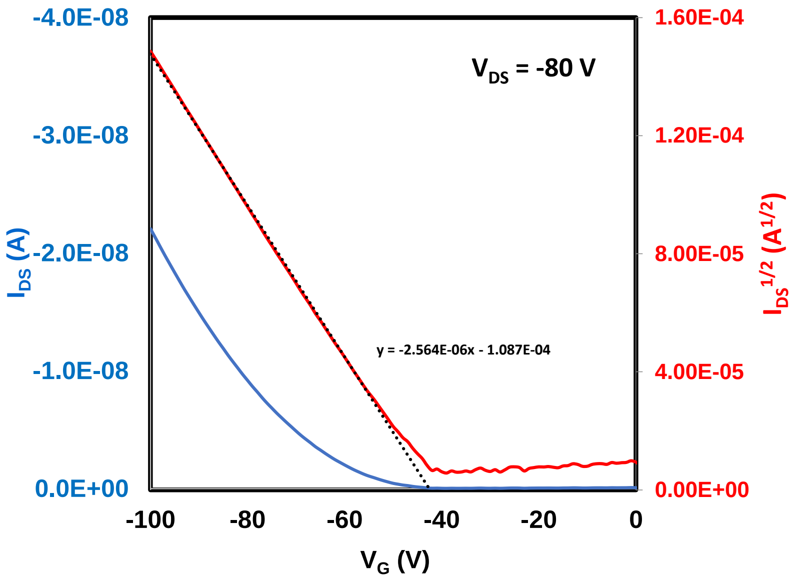

Figure S7. Typical transfer plot for p-type semiconductor **1**. Average mobility (h$^+$= 2.2

Synthesis of Materials

Compound 5 was synthesized as previously described in *Chem. Comm.*, **2018**, 54, 14140-14143

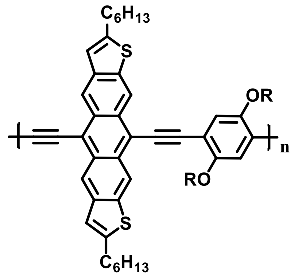

R=2-Ethyl hexyl

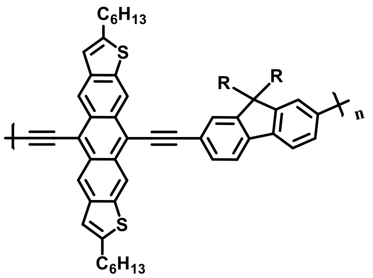

R=Dodecyl

Polymer 1. In a glovebox were combined **6** (16 mg, 0.025 mmol), **7** (9 mg, 0.025 mmol), $Pd(PPh_3)_4$ (2.6 mg, 2.2 μmol), CuI (.49 mg, 2.6 μmol), diisopropylamine (240 μl,)) in toluene (1 ml) in a pressure sealed tube. The contents of the sealed tube were stirred at 80 °C for five days in the absence of light. The reaction mixture was cooled to room temperature and poured in cold methanol (10 ml). The precipitates were filtered and washed with methanol (10 ml) to give **1** as a purple solid (19 mg 76%). ^{1}H NMR (400 MHz, CD_2Cl_2) δ 8.57 – 8.14 (m, 4H), 6.64 (m, J = 93.5, 85.8 Hz, 6H), 3.36 (m, J = 66.2 Hz, 4H), 2.19 (s, 4H), 1.37 (s, 1H), 0.85 (s, 36H), 0.57 (s, 6H), 0.12 (s, 12H). Mn: 15,800 Da; PDI: 3.56.

Polymer 2. In a glovebox were combined **6** (10 mg, 0.017 mmol), **8** (9 mg, 0.017 mmol), $Pd(PPh_3)_4$ (1.968 mg, 1.707 μmol), CuI (0.32 mg, 1.7 μmol), diisopropylamine (240 μl) in toluene (1 ml) in a pressure sealed tube. The contents of the sealed tube were stirred at 80 °C for five days in the absence of light. The reaction mixture was cooled to room temperature and poured in cold methanol (10 ml). The precipitates were filtered and washed with methanol (10 ml) to give **2** as a maroon solid (13 mg 68%). ^{1}H NMR (400 MHz, CD_2Cl_2) δ 8.37 (s, 2H), 8.23 (s, 2H), 7.05 – 6.92 (m, 22H), 6.88 – 6.73 (m, 28H), 6.53 (s, 2H), 2.34 – 2.18 (m, 8H), 1.04 (s, 88H), 0.57 (m, J = 24.1 Hz, 136H), 0.25 (d, J = 12.4 Hz, 61H). Mw: 8,600 Da; PDI: 3.41.

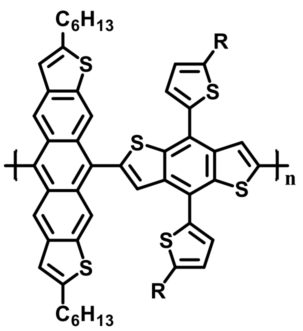

R=Dodecyl

3

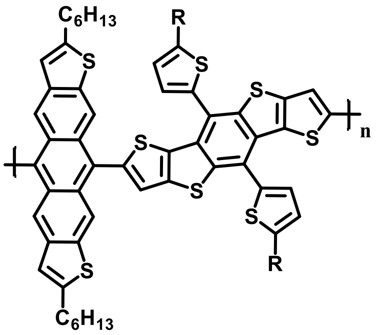

R=Octyl

4

Polymer 3. In a glove box were combined **6** (10 mg, 0.016 mmol), **9** (14 mg, 0.016 mmol), $Pd_2(dba)_3$ (1.5 mg, 1.6 μmol) and *p*(o-Tol)$_3$ (1.5 mg, 4.9 μmol) in 2 mL toluene in a sealable tube. This mixture was stirred at 100 °C for 5 days in the absence of light. The reaction mixture was cooled down to room temperature and precipitation was carried out in methanol. The precipitates were filtered and washed with methanol and hexane and gave 3 and a brick red solid (20 mg, 83%). ^{1}H NMR (400 MHz, $CDCl_3$) δ 8.09 (m, 6H), 7.52 (m, 2H), 6.85 (m, J = 70.3 Hz, 4H), 2.78 (m, J = 65.6 Hz, 8H), 1.56 (s, 12H), 1.28 (m, J = 24.7 Hz, 32H), 0.90 (s, 14H). Mw: 4100 g/mol; PDI: 1.67.

Polymer 4. In a glove box were combined **6** (10 mg, 0.016 mmol), **10** (14 mg, 0.016 mmol), $Pd_2(dba)_3$ $Pd_2(dba)_3$ (1.5 mg, 1.6 μmol) and *p*(o-Tol)$_3$ (1.5 mg, 4.9 μmol) in 2 mL toluene in a sealable tube. This mixture was stirred at 100 °C for 5 days in the absence of light. The reaction mixture was cooled down to room temperature and precipitation was carried out in methanol. The precipitates were filtered and washed with methanol and hexane and gave 4 and a brick red solid (19 mg, 73%). ^{1}H NMR (400 MHz, CD_2Cl_2) δ 6.56 (m, 4H), 6.43 (m, 2H), 6.28 (m, 6H), 1.98 (m, 8H), 0.71 (s, 32H), 0.37 (s, 14H), -0.00 (s, 12H). Mw: 4000 g/mol; PDI: 1.93.

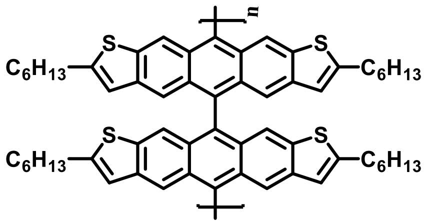

5

Polymer 5. (Ni(cod)2) (17 mg, 0.065 mmol), 2,2′-bipyridine (bpy) (10 mg, 0.065 mmol), and 1,5-cyclooctadiene (cod) (17 μL, 0.065 mmol) in dry DMF (0.25 mL) were stirred at room temperature under N2 atmosphere for 30 min. Then DHADT 5 (10 mg, 0.016 mmol) in dry THF (0.5 mL) was added into the catalyst solution, which was stirred at 100 °C for 5 days. The reaction mixture was poured into the solution cold of MeOH (25mL), and the resultant precipitate was successively purified by reprecipitation from MeOH. Homopolymer 5 was obtained as a yellow solid (9 mg, 90% yield). 1H NMR (400 MHz, CDCl3) δ 9.12 – 7.89 (m, 7H), 7.10 – 6.36 (m, 4H), 3.39 – 2.30 (m, 8H), 1.84 – 1.54 (m, 5H), 1.29 (m, J = 31.7 Hz, 12H), 1.02 – 0.49 (m, 9H).

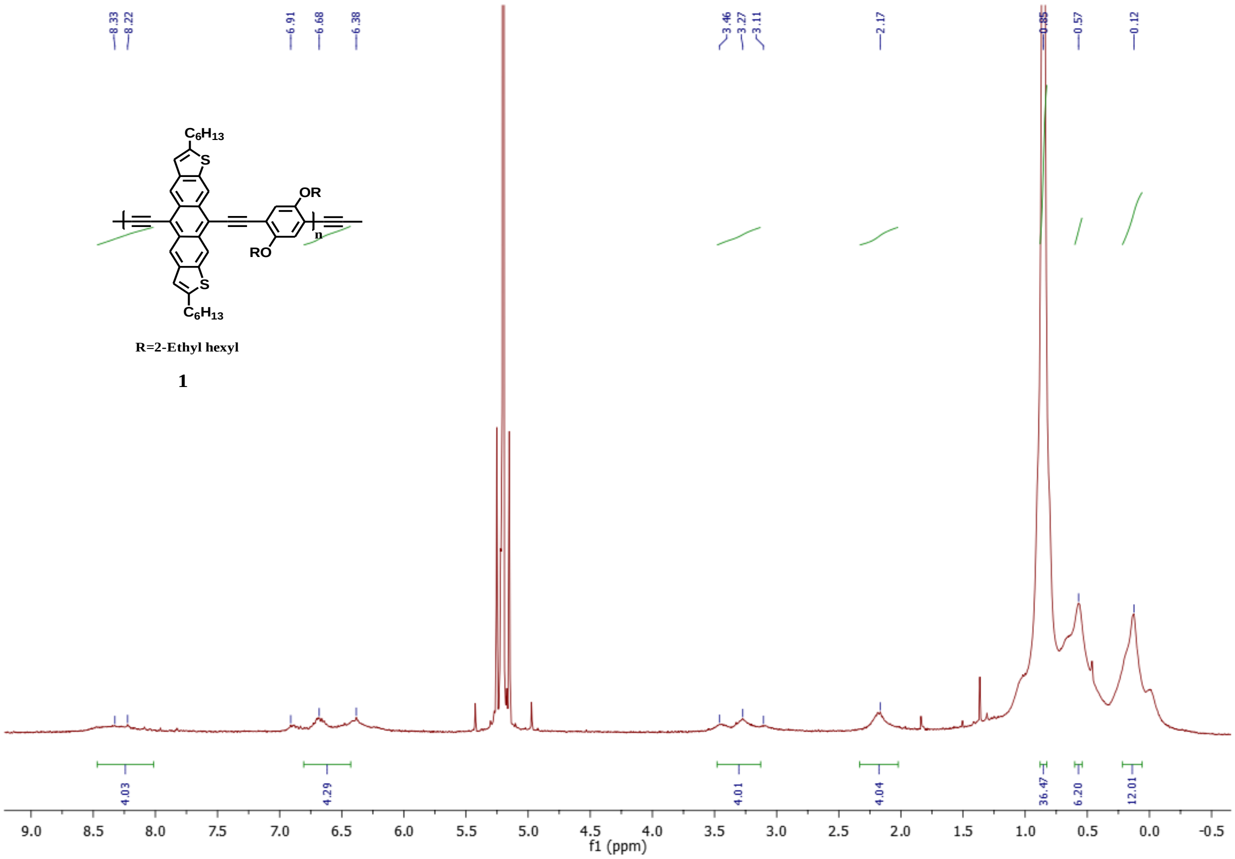
8.33
8.22
6.91
6.68
6.38
3.46
3.27
3.11
2.17
0.85
0.57
0.12
C6H13
S
OR
RO
n
R=2-Ethyl hexyl
1
4.03
4.29
4.01
4.04
36.47
6.20
12.01
9.0 8.5 8.0 7.5 7.0 6.5 6.0 5.5 5.0 4.5 4.0 3.5 3.0 2.5 2.0 1.5 1.0 0.5 0.0 -0.5
f1 (ppm)

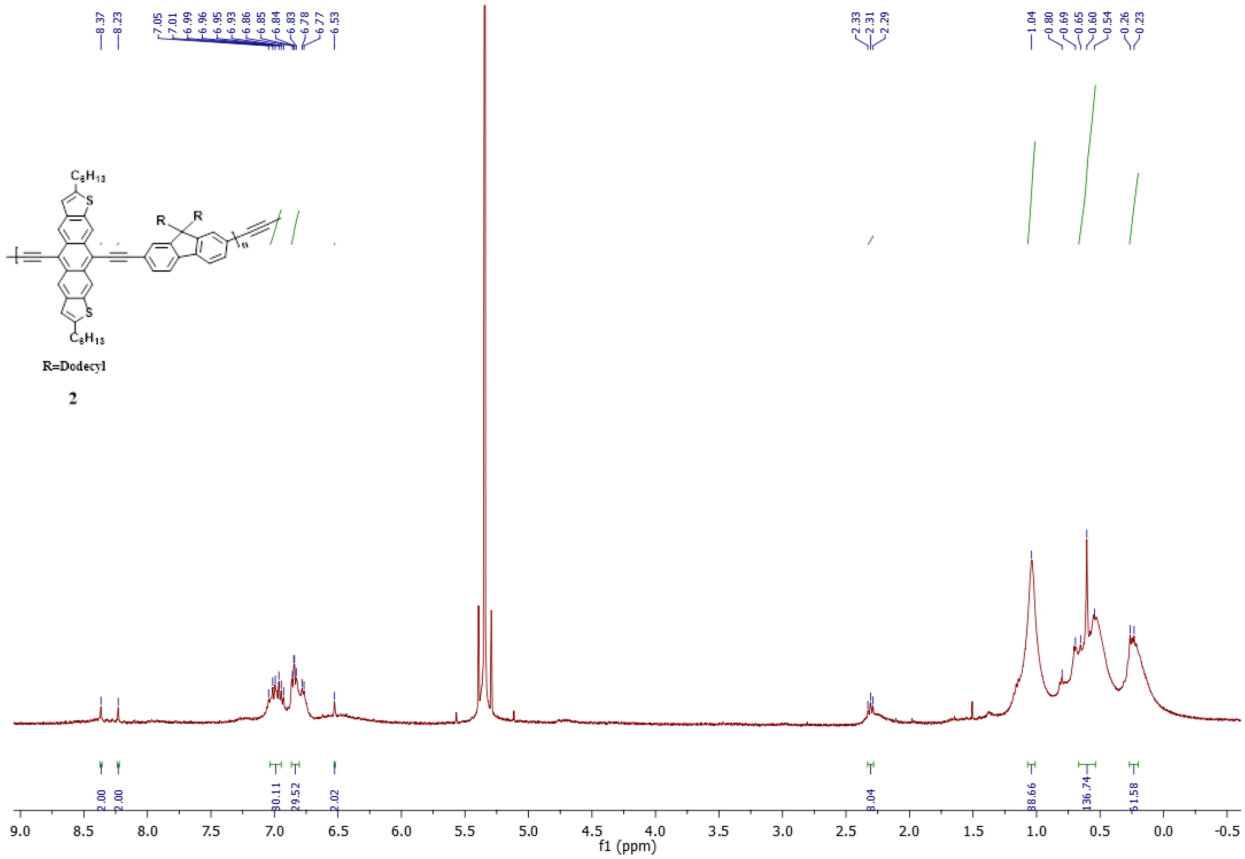

R=Dodecyl
2
f1 (ppm)

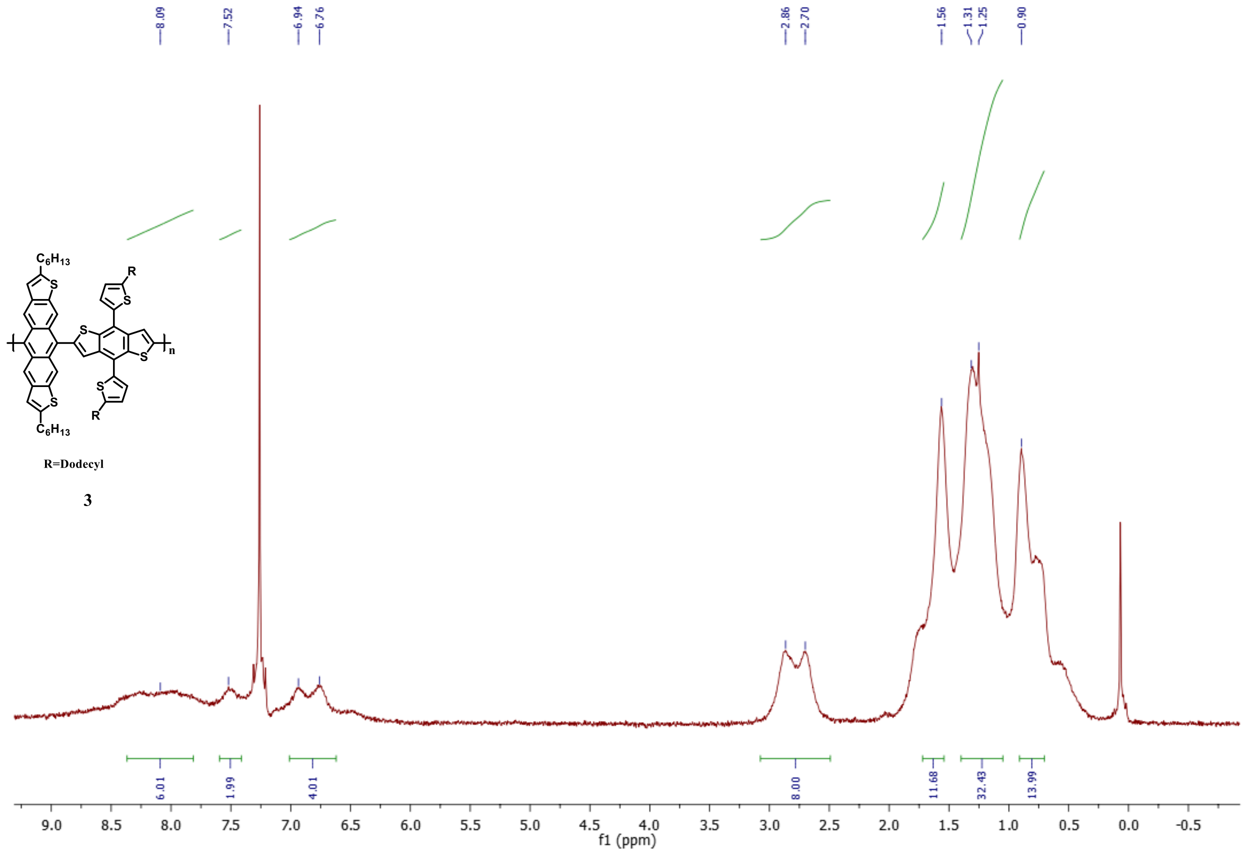
8.09
7.52
6.94
6.76
2.86
2.70
1.56
1.31
1.25
0.90
R=Dodecyl
3
6.01
1.99
4.01
8.00
11.68
32.43
13.99
f1 (ppm)

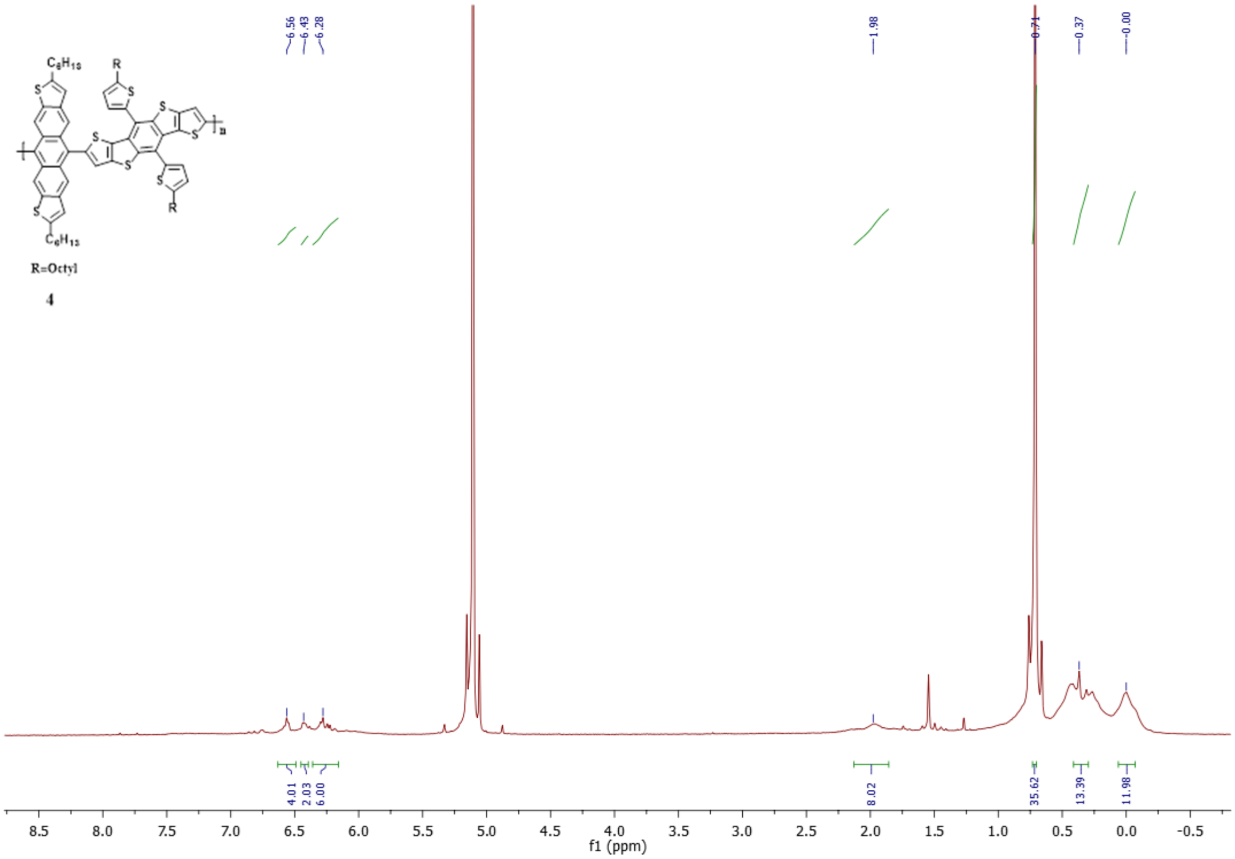

R=Octyl
4
6.56
6.43
6.28
1.98
0.71
0.37
0.00
4.01
2.03
6.00
8.02
35.62
13.39
11.98
f1 (ppm)

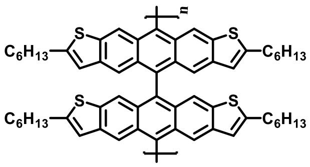
S
S
S
S
C6H13
C6H13
C6H13
C6H13
n

5

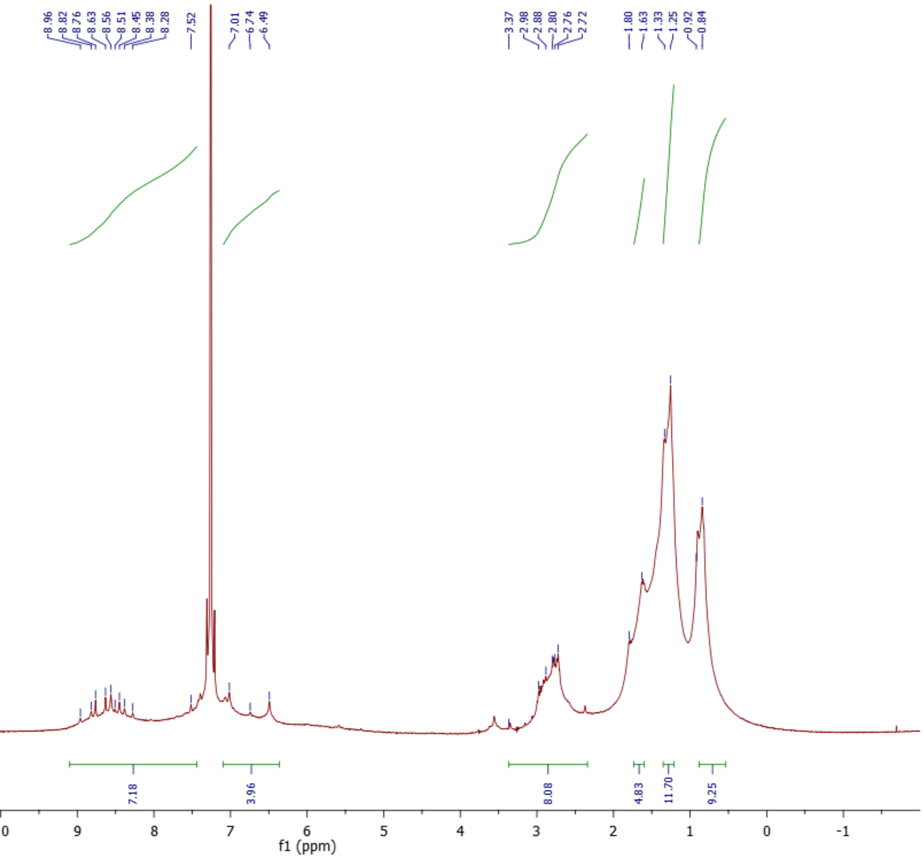
8.96
8.82
8.76
8.63
8.56
8.51
8.45
8.38
8.28
7.52
7.01
6.74
6.49
3.37
2.98
2.88
2.80
2.76
2.72
1.80
1.63
1.33
1.25
0.92
0.84
7.18
3.96
8.08
4.83
11.70
9.25
13
12
11
10
9
8
7
6
5
4
3
2
1
0
-1
f1 (ppm)

Coordinates for DFT Calculated Structure of 1
Job type: Geometry optimization and frequency
Method: B3LYP
Basis set: 6-311G(d,p)
Number of basis functions: 790
Multiplicity: 1
Total Energy: -1230.497005 h
Imaginary Frequencies: 0

C	-3.98517220	-2.58309814	0.00637709
C	-2.84367070	-3.35122190	-0.03596352
C	-1.54629363	-2.76002431	-0.06850322
C	-1.45562776	-1.31457418	-0.04076697
C	-2.64487327	-0.53579712	-0.00718864
C	-3.86390281	-1.15324733	0.01418659
C	-0.37030306	-3.54652328	-0.11619512
C	-0.18318353	-0.68957335	-0.04428334
C	0.99735799	-1.47678377	-0.04352084
C	0.89950571	-2.92274765	-0.06916918
C	2.11094339	-3.67542878	-0.03511560
H	2.07490835	-4.75582198	-0.02157942
C	3.34128498	-3.05996066	0.00398766
C	3.40400933	-1.62674153	0.00711514
C	2.27692868	-0.85481163	-0.01411277
H	-2.94459916	-4.42774440	-0.02766177
H	-2.55874746	0.54292028	0.00796211
H	2.33153561	0.22659371	-0.00191407
C	4.65816125	-3.64990185	0.04543903
C	5.66882155	-2.75196760	0.07417571
S	5.08152856	-1.07530662	0.05451184
H	4.82776644	-4.71982185	0.05136861

C	-6.25259020	-1.97747017	0.08010879
C	-5.36665329	-2.99873824	0.04721688
S	-5.45519116	-0.39037806	0.06553982
H	-5.67264432	-4.03786257	0.04968616
C	-0.07473794	0.72247483	-0.03572440
C	0.07441116	1.92529337	-0.02949235
C	0.27997963	3.32814254	-0.02154898
C	-0.79924235	4.21569599	-0.02963818
C	1.60052768	3.85181956	-0.00511477
C	-0.60325376	5.59825571	-0.02176033
H	-1.81214363	3.83485921	-0.04253553
C	1.78722480	5.22798512	0.00254896
C	0.69469837	6.10369274	-0.00564390
H	2.78434382	5.64635522	0.01507332
H	0.88191760	7.16872376	0.00074537
O	2.60743614	2.93160731	0.00213649
C	3.94853631	3.39759232	0.01789727
H	4.17584423	3.99531900	-0.87200115
H	4.15726478	3.98839722	0.91692782
H	4.57388703	2.50641992	0.02096513
O	-1.74102598	6.35731755	-0.03099383
C	-1.60421860	7.76895676	-0.02386659
H	-2.61770259	8.16701437	-0.03326593
H	-1.08767621	8.12018599	0.87723190
H	-1.06828854	8.12732687	-0.91071723
C	-7.74559888	-2.04824898	0.12358893
H	-8.14332728	-1.56187085	1.01981879
H	-8.19505896	-1.55348760	-0.74312701
H	-8.07238012	-3.08975238	0.12821355
C	7.14022646	-3.01480668	0.11703471
H	7.64927730	-2.58529114	-0.75158099
H	7.59851258	-2.58154066	1.01165698

H	7.32963334	-4.08980078	0.12477180
C	-0.48082037	-5.05175591	-0.19876766
H	-0.59620582	-5.50517872	0.79313276
H	-1.33887191	-5.35576865	-0.79858543
H	0.39522550	-5.49953319	-0.66435597

Coordinates for DFT Calculated Structure of 2
Job type: Geometry optimization and frequency
Method: B3LYP
Basis set: 6-311G(d,p)
Number of basis functions: 934
Multiplicity: 1
Total Energy: -2301.0104903 h
Imaginary Frequencies: 0

C	-3.44159825	4.12437330	0.00727600
C	-4.37798232	3.11686323	-0.03376800
C	-3.99814429	1.74227212	-0.06894400
C	-2.58347519	1.42624210	-0.04228000
C	-1.62989512	2.48069918	-0.01063800
C	-2.04863715	3.78119827	0.01153000
C	-4.95667136	0.70230705	-0.11800301
C	-2.16158715	0.07125400	-0.04451200
C	-3.12424023	-0.97167907	-0.04121000
C	-4.53607333	-0.64825705	-0.06944201
C	-5.47224437	-1.72351912	-0.03643300
H	-6.53277945	-1.51331811	-0.02774600
C	-5.06161837	-3.03652222	0.00644200
C	-3.65664127	-3.32676124	0.01420400
C	-2.71778819	-2.33376017	-0.00745000
H	-5.42479638	3.38635124	-0.02082600
H	-0.57797004	2.22817516	0.00316900
H	-1.65982012	-2.55958418	0.00821300

C	-5.85328940	-4.24249630	0.04818100
C	-5.12635437	-5.38237240	0.08174001
S	-3.37785424	-5.06903437	0.06659200
H	-6.93649650	-4.24017731	0.05080200
C	-2.48971418	6.26965246	0.08069801
C	-3.63656726	5.55387837	0.05004200
S	-1.04642908	5.23361638	0.06146000
H	-4.61518033	6.01826141	0.05535000
C	-0.77937406	-0.24068302	-0.03780000
C	0.40441403	-0.50873804	-0.03407300
C	-2.32588317	7.75522154	0.12517401
H	-1.78215613	8.07119556	1.02099308
H	-1.76819113	8.12249760	-0.74204205
H	-3.30342024	8.24086661	0.13155101
C	-5.61852341	-6.79360948	0.12637801
H	-5.27109638	-7.36614852	-0.73932605
H	-5.26618638	-7.31204354	1.02361408
H	-6.70992651	-6.81086548	0.12994001
C	-6.43089648	1.02484307	-0.20369701
H	-6.89835949	1.05361107	0.78796306
H	-6.60966446	1.98770214	-0.67886305
H	-6.96728149	0.28385902	-0.79642206
C	2.22088816	-2.16332316	-0.03309200
C	1.78979513	-0.82006806	-0.02948900
C	2.75594720	0.21026102	-0.02118200
C	4.10056929	-0.11050701	-0.01651100
C	4.51714133	-1.45567511	-0.01978700
C	3.57209026	-2.48281318	-0.02827500
H	1.47741811	-2.95108121	-0.03998600
H	2.42437517	1.24241809	-0.01877800
H	3.88014628	-3.52261725	-0.03100400
C	5.30490937	0.83136406	-0.00769400

C	6.46087749	-0.16874301	-0.00457800
C	7.82415556	0.08202801	0.00027700
C	8.73774261	-0.98318707	-0.00046900
C	8.24399659	-2.29400917	-0.01095900
C	6.87614452	-2.55880919	-0.01619600
C	5.98050843	-1.49100411	-0.01219200
H	8.19770458	1.10216708	0.00282800
H	8.94607467	-3.12153223	-0.01695100
H	6.52216246	-3.58406726	-0.02690500
C	5.33269837	1.71876212	-1.27095109
H	4.46562132	2.38465917	-1.29449109
H	6.23343843	2.33873917	-1.28606909
H	5.32106240	1.11029108	-2.17773616
C	5.31977036	1.71223312	1.26031509
H	4.45231832	2.37781117	1.27863809
H	5.29918139	1.09908908	2.16379916
H	6.22013142	2.33233617	1.28767409
C	10.22369175	-0.71670905	0.03399800
H	10.49299376	0.12682601	-0.60720404
H	10.55740876	-0.47235203	1.04869308
H	10.79297180	-1.58762512	-0.29748802

Coordinates for DFT Calculated Structure of 3
Job type: Geometry optimization
Method: B3LYP
Basis set: 6-311G(d,p)
Number of basis functions: 1062
Multiplicity: 1
Total Energy: -3968.9055022 h
Imaginary Frequencies: 0

C	4.04537431	-0.63695542	-1.02544138
C	4.31680254	-0.28175847	0.32552284
C	3.25480558	0.03053879	1.20099017

C	1.96399518	-0.03903471	0.66926512
C	1.69351813	-0.39474233	-0.68196728
C	2.75435943	-0.70577011	-1.55771202
C	6.47248827	-0.71899814	-0.44529370
C	5.72555591	-0.34577621	0.61959397
H	7.54454317	-0.84173200	-0.48609027
H	6.14117209	-0.12780012	1.59271601
C	0.28597228	-0.33117885	-0.97777514
C	-0.48735055	0.04198945	0.07365138
S	0.47593313	0.34777094	1.52623645
H	-0.12751242	-0.54967006	-1.95230430
S	5.53186783	-1.02534812	-1.88670273
C	2.53346086	-1.08259022	-2.96516452
C	2.99618244	-0.46342181	-4.09512497
C	2.61972625	-1.12040713	-5.30198360
C	1.87411295	-2.24842001	-5.10630352
S	1.62377243	-2.51996207	-3.39939037
H	3.57799846	0.44838616	-4.06144517
H	2.89052216	-0.76328963	-6.28785166
C	3.47591868	0.41022699	2.60757552
C	2.99997195	-0.19664051	3.73868009
C	3.37715567	0.46289104	4.94389571
C	4.13680759	1.58100692	4.74631208
S	4.40007288	1.83983826	3.03914791
H	2.40563132	-1.10036704	3.70744477
H	3.09460017	0.11561208	5.92995904
C	4.70260802	2.51684472	5.77082581
H	5.79438091	2.56769967	5.71860416
H	4.31908566	3.53429679	5.64875467
H	4.43039607	2.17455060	6.77130604
C	1.30949228	-3.18274973	-6.13284824
H	1.69903427	-4.19867466	-6.01733631

H	0.21833760	-3.23971517	-6.07558512
H	1.57510729	-2.83402502	-7.13289248
C	-3.70459614	3.98572414	-0.72279570
C	-4.51482032	2.91775349	-0.41549375
C	-3.97915383	1.62376657	-0.14181710
C	-2.53486668	1.46082385	-0.17830170
C	-1.71311163	2.57972083	-0.51050275
C	-2.28279561	3.79194117	-0.77262262
C	-4.80400648	0.51802461	0.16766311
C	-1.96090910	0.20624274	0.11234970
C	-2.77887989	-0.88899155	0.45912411
C	-4.22052575	-0.72807901	0.49447367
C	-5.01608944	-1.84931755	0.87290740
H	-6.09140292	-1.75047077	0.93015989
C	-4.44844373	-3.06283112	1.18712298
C	-3.02063333	-3.20081898	1.12626011
C	-2.21046249	-2.15859554	0.77706321
H	-5.58233520	3.08087226	-0.36860546
H	-0.64017374	2.44898071	-0.54750788
H	-1.13609727	-2.27687724	0.73751984
C	-5.08413422	-4.29403294	1.58997169
C	-4.22471535	-5.31232827	1.82145375
S	-2.53181293	-4.84118560	1.56223098
H	-6.15687876	-4.39897834	1.69768331
C	-3.01236121	6.15643494	-1.28945442
C	-4.06511639	5.35062134	-1.02282986
S	-1.46035166	5.29756312	-1.19210251
H	-5.08918233	5.70350467	-1.03599591
C	-3.02485498	7.61059798	-1.63726803
H	-2.45930887	8.20067650	-0.90928978
H	-2.57867370	7.79058164	-2.62039315
H	-4.05035139	7.98416553	-1.65526206

C	-4.53992511	-6.71065174	2.24703975
H	-4.18846292	-7.44141938	1.51188956
H	-4.06496583	-6.95466918	3.20247403
H	-5.61835666	-6.83466226	2.36155007
C	-6.31042991	0.65021223	0.16934586
H	-6.70353812	0.77259932	1.18540725
H	-6.65188433	1.49852796	-0.41914216
H	-6.78414822	-0.23455585	-0.25880919

Coordinates for DFT Calculated Structure of 4
Job type: Geometry optimization
Method: B3LYP
Basis set: 6-311G(d,p)
Number of basis functions: 1216
Multiplicity: 1
Total Energy: -4957.1153304h
Imaginary Frequencies: 0

C	-5.78912423	3.38415449	-1.15669276
C	-6.45922741	2.21108024	-0.89591954
C	-5.76446081	0.98162857	-0.69673526
C	-4.31412122	0.99459041	-0.75869962
C	-3.63881480	2.21913278	-1.04290543
C	-4.35554159	3.36549146	-1.23416188
C	-6.45152211	-0.22652891	-0.43568701
C	-3.59204312	-0.19751674	-0.54026848
C	-4.27164567	-1.39663225	-0.24024867
C	-5.72345687	-1.41048191	-0.17628498
C	-6.36949482	-2.63700265	0.16132696
H	-7.44650435	-2.67302263	0.24734948
C	-5.65515036	-3.78702871	0.40444982
C	-4.22281995	-3.75123575	0.31188452

C	-3.55048052	-2.60395318	0.00361315
H	-7.53809350	2.23631653	-0.82834163
H	-2.55876976	2.22101391	-1.10029741
H	-2.47078051	-2.59003227	-0.05750510
C	-6.13168521	-5.10290037	0.75686891
C	-5.15300683	-6.02148220	0.92173666
S	-3.53371951	-5.34055110	0.65650611
H	-7.18174517	-5.33974505	0.87804230
C	-5.37193591	5.64787163	-1.61858386
C	-6.31574798	4.70878242	-1.38091189
S	-3.72714407	4.97828599	-1.58519174
H	-7.37474294	4.93596527	-1.36335702
C	-5.56490881	7.10537442	-1.89104743
H	-5.05918791	7.72266090	-1.14193940
H	-5.16343480	7.38585069	-2.86988654
H	-6.62781333	7.35333418	-1.87528593
C	-5.29071133	-7.46608523	1.28207871
H	-4.87265674	-8.11373352	0.50506057
H	-4.76783614	-7.69701565	2.21549024
H	-6.34391873	-7.72314986	1.40924795
C	-7.96364319	-0.23697700	-0.42099813
H	-8.36111896	0.07020345	0.55361774
H	-8.37324739	0.44158691	-1.16952408
H	-8.36754572	-1.22239929	-0.64466118
C	3.76505876	0.42420120	1.43899005
C	4.54633972	0.16103808	0.27453371
C	3.90712030	-0.15000821	-0.94115810
C	2.51264979	-0.18618725	-0.93736912
C	1.73240751	0.07659569	0.22699680
C	2.37116506	0.38809806	1.44277889
C	6.21566686	0.57228315	1.90257582
C	5.94380125	0.26264190	0.58543577

C	0.33314520	-0.02453446	-0.08158497
C	0.06161318	-0.33294065	-1.40068592
S	1.51836288	-0.52822726	-2.36241221
S	4.75920090	0.76826568	2.86343852
C	1.59900301	0.66603052	2.67260107
C	1.08165153	-0.22282477	3.57419439
C	0.38362412	0.39861601	4.65014408
C	0.36240172	1.76307859	4.57728830
S	1.21839496	2.30881919	3.15799468
H	1.19875428	-1.29339011	3.46688523
H	-0.08993451	-0.15115921	5.45390462
C	4.67777977	-0.42560285	-2.17239875
C	5.20264087	0.46544517	-3.06743004
C	5.89027476	-0.15375009	-4.15141665
C	5.89601454	-1.51907319	-4.09145730
S	5.03914498	-2.06811034	-2.67399213
H	5.09576750	1.53608714	-2.95074595
H	6.36481367	0.39794964	-4.95337267
C	6.51465783	-2.49039001	-5.05086198
H	7.26782612	-3.11826337	-4.56565624
H	5.76760878	-3.15489152	-5.49511610
H	7.00346875	-1.94601940	-5.86141063
C	-0.27532223	2.73614493	5.52219293
H	0.45853132	3.42024735	5.95865626
H	-1.03886964	3.34275333	5.02640783
H	-0.75616003	2.19398076	6.33892039
C	-1.32169236	-0.42638670	-1.70939672
C	-2.11131593	-0.18740986	-0.61557327
S	-1.14631148	0.15463389	0.82220881
H	-1.72652753	-0.65796563	-2.68518455
C	7.60023511	0.66511738	2.21081145
C	8.38647619	0.42371123	1.11831340

S	7.42713371	0.08093770	-0.31670957
H	8.00116313	0.89817299	3.18830253
C	9.88255764	0.42953300	1.04321857
H	10.27729894	-0.54365851	0.73563218
H	10.25057025	1.17433133	0.33092355
H	10.30161776	0.66688057	2.02302670

Coordinates for DFT Calculated Structure of 5
Job type: Geometry optimization
Method: B3LYP
Basis set: 6-311G(d,p)
Number of basis functions: 706
Multiplicity: 1
Total Energy: -2975.4929827h
Imaginary Frequencies: 0

C	2.44672400	0.44369900	0.90975100
C	1.21050000	0.21806500	1.58862000
C	1.20780300	0.21533200	3.04182600
C	2.42243000	0.43459500	3.75067200
C	0.00115000	-0.00037200	0.88531800
C	0.00426200	-0.00397200	3.72042100
C	-1.20079600	-0.22140000	3.04388200
C	-1.20681500	-0.22014700	1.59066900
C	-2.44475700	-0.44310000	0.91408400
H	-2.46178100	-0.44427400	-0.16945300
C	-3.58893600	-0.65159100	1.63960300
C	-3.59742000	-0.65559900	3.08226300
C	-2.41380700	-0.44239700	3.75494100

H	2.46107400	0.44804200	-0.17383000
H	2.40291500	0.42934500	4.83739700
H	-2.39175300	-0.44014100	4.84162700
C	3.59251400	0.65056900	1.63317400
C	3.60435000	0.65034700	3.07583200
C	4.93115000	0.89044900	3.59727700
H	5.15659800	0.92967800	4.65691000
S	5.21483900	0.94630800	0.99375100
C	5.85958000	1.06083800	2.62839500
C	-5.85392800	-1.06342500	2.63888400
C	-4.92310600	-0.89663200	3.60609900
S	-5.21290100	-0.94457300	1.00309200
H	-5.14610700	-0.93885200	4.66613400
C	-0.44457900	2.41995000	-3.48407200
C	-0.22263700	1.20452900	-2.77085200
C	-0.22114100	1.21102700	-1.31773000
C	-0.44367500	2.44622600	-0.64302000
C	-0.00541400	0.00175200	-3.44996200
C	-0.00048600	0.00056800	-0.61528600
C	0.21821800	-1.20928500	-1.31945400
C	0.21436700	-1.20155200	-2.77258300
C	0.43396400	-2.41628900	-3.48767500
H	0.42813500	-2.39422100	-4.57350500
C	0.64670300	-3.57894000	-2.79623800
C	0.65482600	-3.61017200	-1.35409700
C	0.44411000	-2.44491600	-0.64668000
H	-0.44274300	2.39884100	-4.56993600
H	-0.44451600	2.46309400	0.44110300
H	0.44916100	-2.46279900	0.43740200
C	-0.65400200	3.58213200	-2.79083400
C	-0.65656400	3.61217300	-1.34863200
C	-0.89737500	4.94709600	-0.84799700

H	-0.93878300	5.18687300	0.20831700
S	-0.94756700	5.19392300	-3.45563100
C	-1.06514800	5.86230600	-1.82942200
C	1.06389300	-5.85948100	-1.83832000
C	0.89890800	-4.94526200	-0.85549900
S	0.93876800	-5.18998300	-3.46350900
H	0.94451200	-5.18588800	0.20044900
H	-0.00786900	0.00209200	-4.53746400
H	-6.91084400	-1.25507100	2.76953500
H	6.91669000	1.25270000	2.75714500
H	-1.25674500	6.92118700	-1.71634900
H	1.25710700	-6.91824000	-1.72687200
H	0.00553800	-0.00588000	4.80801500
